# Constitutive activation of mTORC1 signaling induced by biallelic loss-of-function mutations in *SZT2* underlies a discernible neurodevelopmental disease

**DOI:** 10.1371/journal.pone.0221482

**Published:** 2019-08-20

**Authors:** Yuji Nakamura, Kohji Kato, Naomi Tsuchida, Naomichi Matsumoto, Yoshiyuki Takahashi, Shinji Saitoh

**Affiliations:** 1 Department of Pediatrics and Neonatology, Nagoya City University Graduate School of Medical Sciences, Nagoya, Japan; 2 Department of Pediatrics, Nagoya University Graduate School of Medicine, Nagoya, Japan; 3 Department of Human Genetics, Yokohama City University Graduate School of Medicine, Yokohama, Japan; Medizinische Universitat Innsbruck Department fur Kinder- und Jugendheilkunde, AUSTRIA

## Abstract

There have been increasing number of reports of *SZT2*-related neurological diseases, the main symptoms of which are epilepsy, developmental delay, macrocephaly and a dysmorphic corpus callosum. SZT2 functions as a regulator of mechanistic target of rapamycin complex 1 (mTORC1) signaling in cultured human cell lines and mouse tissues. However, it remains to be determined whether mutations in *SZT2* in human patients alter mTORC1 signaling. In this study, we aimed to investigate the functional consequence of biallelic *SZT2* variants in Epstein-Barr virus-induced lymphoblastoid cell lines (LCLs) established from two patients with a typical *SZT2*-related neurodevelopmental disease. Increased phosphorylation of S6 kinase and S6 was identified in patient-derived cell lines under amino acid-starved condition, suggestive of constitutive hyperactivation of mTORC1 signaling. This result was validated by constitutive lysosomal localization of mTOR in patients’ LCLs. Furthermore, patients’ LCLs display an excessive response to slight amino acid stimulation. Our data suggest the loss-of-function nature of *SZT2* mutations in the patients, and consequent hyperactivation of mTORC1 signaling in response to both amino acid starvation and stimulation in their LCLs. By these functional analyses, the pathogenicity of newly identified *SZT2* variants can be determined, allowing for more detailed characterization of genotype-phenotype correlations.

## Introduction

*Seizure threshold 2* (*SZT2*), containing 71 exons located in chromosome 1p34.2, was first identified as a gene that conferred low seizure threshold to knockout mice [1]. Biallelic variations in *SZT2* have been demonstrated to cause a characteristic clinical entity with epilepsy, developmental delay, macrocephaly and a dysmorphic corpus callosum [2–6]. Since the first report in 2013 [7], an additional 13 cases from 10 families with *SZT2* variants have been reported [8–15]. Four of the 15 patients carried biallelic null variants that were assumed to cause complete loss of SZT2 function [7, 13, 15]. The remaining 11 patients carried missense, in-frame deletion or intronic (outside canonical ±1 or 2 splice sites) variants in at least either allele, which were assumed to lose partial or full SZT2 function.

Recent reports have shown that SZT2 forms a protein complex, KICSTOR (consisting of KPTN, ITFG2, C12orf66, and SZT2), and functions as a regulator of mechanistic target of rapamycin complex 1 (mTORC1) signaling in cultured human cell lines and in mice [16, 17]. In this signaling pathway, sestrin2 and CASTOR1 directly sense leucine and arginine, respectively, and their inhibition of GATOR2 activity (consisting of Mios, WDR24, WDR59, Seh1L, and Sec13) is itself negatively regulated by these amino acids [18, 19]. GATOR2, in turn, negatively regulates GATOR1 (consisting of DEPDC5, Nprl2, and Nprl3) [20], which in turn inhibits mTORC1 signaling interacting with KICSTOR [16, 17]. In brief, KICSTOR regulates the kinase activity of mTORC1 in response to amino acid input [16, 17]. When mTORC1 is activated with amino acid stimulation, S6 kinase (S6K), a downstream substrate, is phosphorylated, which in turn phosphorylates downstream S6, and eventually this cascade leads to cell growth and proliferation [20, 21]. Hyperactivation of the mTORC1 signaling pathway is well-recognized in the etiology of neurological diseases including epilepsy, developmental delay and macrocephaly [2–6]. SZT2 deficiency is assumed to induce hyperactivation of this pathway, and consequently cause such neurological symptoms in humans [16, 17].

Despite SZT2 functions being elucidated, it remains to be demonstrated whether the *SZT2* variants affect protein function and result in hyperactivation of mTORC1 signaling in human patients. In this study, we aimed to assess the functional consequence of the *SZT2* variants in two patients with a typical *SZT2*-related neurodevelopmental disease. Based on the fact that *SZT2* is expressed ubiquitously, we hypothesized that decreased SZT2 function can be demonstrated using patients’ peripheral blood cells [16, 17]. We therefore conducted functional characterization via immunoblotting and immunofluorescence using Epstein-Barr virus-induced lymphoblastoid cell lines (LCLs) established from the two patients.

## Materials and methods

### Patients

We evaluated two patients with *SZT2* variants who have already been described, and three healthy control subjects. Patient 1 was 4-year-old girl with c.8596dup (p.Tyr2866Leufs*42; NM_015284.3) and c.2930-17_2930-3delinsCTCGTG [10]. Patient 2 was 2-year-old boy with c.3947dup (p.Glu1317Glyfs*4) and c.2929+1G>A [13]. They shared common symptoms with other patients with *SZT2* variants, namely developmental delay, intellectual disability, epilepsy and dysmorphic corpus callosum. Patient 1 was compound heterozygote of a frameshift variant and an intronic variant. The intronic variant was considered pathogenic because all the transcripts from the intronic variant allele examined were aberrant, but the mild phenotypic severity of the patient, with walking unassisted, communicating with others and controllable epilepsy, indicated residual partial SZT2 function [10]. Patient 2 was a compound heterozygote of a frameshift variant and a canonical splice-site variant which was demonstrated to lead to exon skipping. These variants were assumed to completely disrupt gene function, and consistent with this, patient 2 showed a severe phenotype, being bedridden, uncommunicative and suffering intractable epilepsy [13]. The Ethical Committee for the Study of Human Gene Analysis at Nagoya City University Graduate School of Medical Sciences approved this work. Written informed consent was obtained from their guardians.

### Cell lines and culture

Epstein-Barr virus-induced LCLs were established from peripheral blood using a standard method [22]. Cells were cultured in DMEM (25 mM glucose; Sigma-Aldrich, Tokyo, Japan) supplemented with 10% fetal bovine serum (FBS), 100 U/ml penicillin,100 mg/ml streptomycin, and 29.2mg/mL L-glutamine. All cell lines were maintained at 37°C and 5% CO2.

### Amino acid starvation and re-stimulation

For amino acid starvation, cells were rinsed and incubated with amino acid-free DMEM (25 mM glucose; FUJIFILM Wako Pure Chemical Corporation, Tokyo, Japan) supplemented with 10% dialyzed FBS (GE Healthcare, Tokyo, Japan) for 1 h. After the treatment, cells were rinsed and stimulated with amino acid-containing DMEM supplemented with 10% FBS for 10 min and 1 h. The formulation of amino acid-containing DMEM was as follows (in g/L): L-Arginine, 0.084; L-Cystine, 0.0626; L-Glutamine, 0.584; Glycine, 0.03; L-Histidine, 0.042; L-Isoleucine, 0.105; L-Leucine, 0.105; L-Lysine, 0.146; L-Methionine, 0.03; L-Phenylalanine, 0.066; L-Serine, 0.042; L-Threonine, 0.095; L-Tryptophan, 0.016; L-Tyrosine, 0.10379; L-Valine, 0.094.

### Cell lysis and immunoblotting

For protein extracts used in immunoblotting, cells were rinsed twice with ice-cold phosphate-buffered saline (PBS) and lysed immediately with sodium dodecyl sulfate (SDS) sample buffer (0.125 mol/L Tris–HCl, pH 6.8, 10% glycerol, 4% SDS, 0.01% bromophenol blue, 5% 2-mercaptoethanol), and subsequently boiled for 5 min. Proteins in the lysates were subjected to SDS-polyacrylamide gel electrophoresis (5–20%), followed by transfer to polyvinylidene difluoride membranes and blocked with PBS with 0.1% Tween containing 5% nonfat milk powder for 1 h at room temperature. Membranes were incubated with primary antibodies overnight at 4°C, followed by 1 h incubation with horseradish peroxidase–conjugated secondary antibody at room temperature. All images were acquired from an Amersham Imager 600 (GE Healthcare) and analyzed using Image J software. The primary antibodies used were S6 (CST #2217; 1:1,000), S6 phosphorylated (p) S240/S244 (CST #5364; 1:1,000), S6K (CST #9202; 1:1,000), S6K pT389 (CST #9234; 1:1,000), AKT (CST #4691; 1:1,000), AKT pS473 (CST #4060; 1:2,000), GAPDH (CST #5174; 1:10,000).

### Immunofluorescence

After amino acid starvation and re-stimulation, cells were rinsed twice with ice-cold PBS and mounted on dishes with glass bottom (Greiner Bio-One, Stuttgat, Germany) using SmearGell (GenoStaff, Tokyo, Japan). The embedded samples were fixed with 4% paraformaldehyde/PBS for 15 min at room temperature. They were then rinsed twice with PBS, and permeabilized with 0.5% Triton X-100 in PBS for 6 min. After rinsing twice with PBS, the samples were blocked for 40 min with Blocking-One (Nacalai Tesque, Kyoto, Japan), and then incubated with primary antibodies in Blocking-One overnight at 4°C, rinsed twice with PBS, and incubated with Alexa Fluor 488 and 594-conjugated secondary antibodies (Abcam, Cambridge, UK) for 30 min at room temperature. The cells were washed with PBS and DAPI (Sigma-Aldrich) for nuclear staining, and then rinsed with 0.1% Triton X-100. Images were acquired on a spinning disk confocal super-resolution microscope (SpinSR10, Olympus, Tokyo, Japan) with a 100 X oil immersion objective lens (Olympus). To quantify co-localization, Pearson’s correlation coefficients were calculated from approximately 50–100 cells per sample using Cellsens imaging software (Olympus) with manually set thresholds. All the images were obtained and analyzed in a single setting. The primary antibodies used were mTOR (CST #2983; 1:400) and LAMP1 (Santa Cruz Biotechnology #SC-20011; 1:400).

### Statistical analysis

The data in the figures represent mean ± SEM of at least three independent experiments. Statistical analysis of data was performed using SPSS software (SPSS, Version 25.0, Chicago, USA). In case of multiple comparisons, statistical significance was calculated by one-way analysis of variance followed by Tukey’s post hoc test. For single comparisons, a standard two-tailed *t*-test was used. Results were considered statistically significant at a *p-* value of < 0.05.

## Results

### Immunoblotting

**Fig 1:**
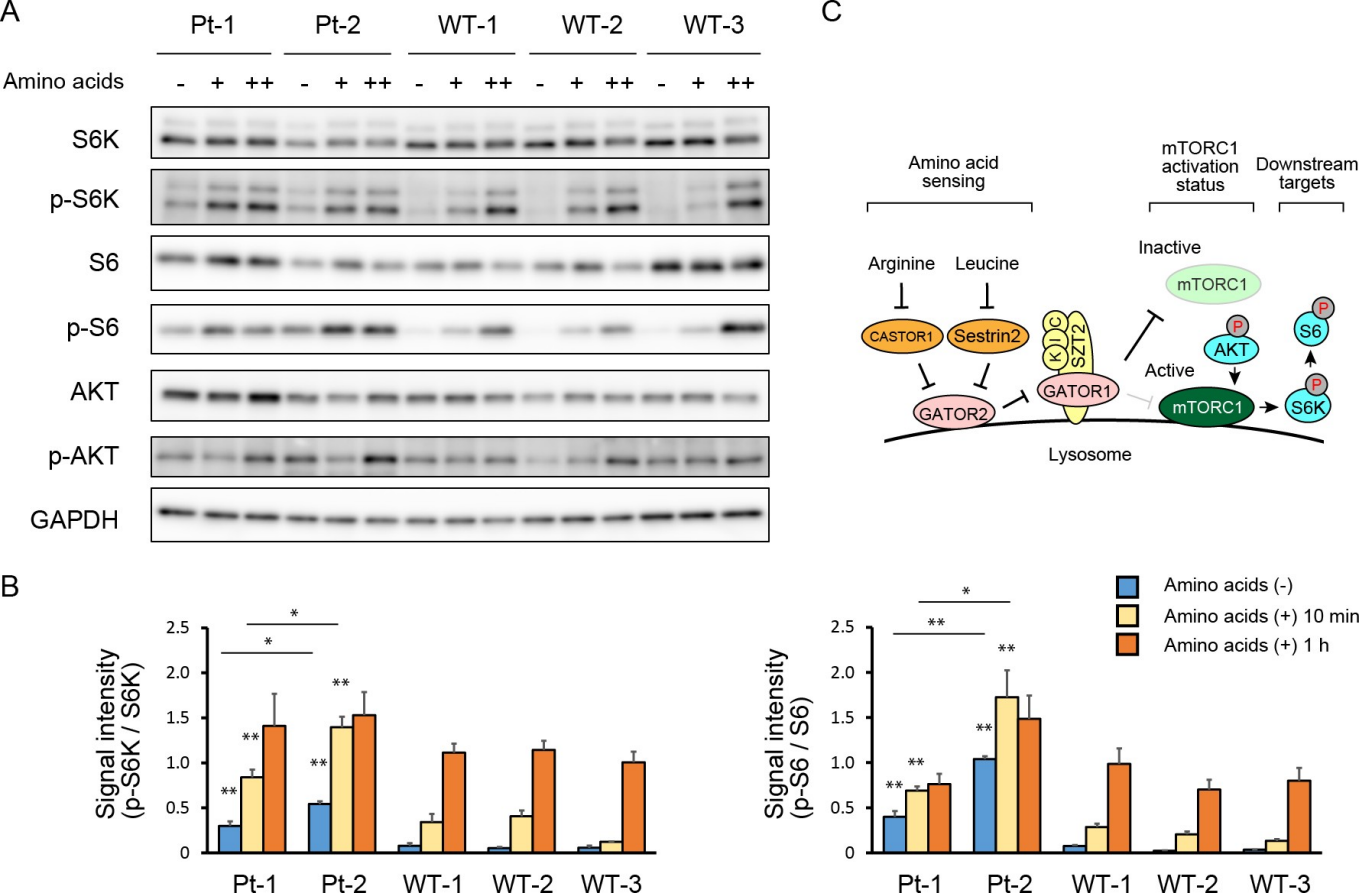
Hyperactivation of mTORC1 signaling under both starved and re-stimulated conditions in the patients. (A) Levels and phosphorylation status of the indicated proteins in the patients and wild-type subjects were determined by immunoblotting. Cells were re-stimulated with amino acids for 10 min (lane +) and 1 h (lane ++). GAPDH was used as a loading control. WT, wild-type. (B) Quantification of the ratio of p-S6K to S6K and p-S6 to S6 bands. Phosphorylation levels of patient 1 and 2 were significantly higher than those of controls under starved and 10 min stimulated conditions, whereas no significant difference after 1 h. Comparing two patients, patient 2 tended to be more activated than patient 1. *p < 0.05. ** *p* < 0.01. (C) Simplified schema of the amino acid-sensing pathway upstream of mTORC1 and the downstream targets. K, I and C represent KPTN, ITFG2 and C12orf66, respectively. They form KICSTOR complex with SZT2. P denotes phosphorylation.

To investigate whether the *SZT2* variants could alter the mTORC1 signaling in the patients’ LCLs, we treated both cell lines with amino acid-free medium and measured phosphorylation levels of S6K and S6, the downstream substrates of mTORC1. Immunoblotting analysis revealed that phosphorylation levels of S6K and S6 were highly increased in the patients’ cell lines, indicating constitutive hyperactivation of mTORC1 signaling under amino acid deprivation conditions (Fig 1A and 1B). In contrast, phosphorylated (p-) S6K and p-S6 were at lower levels in the control cell lines, indicating suppressed mTORC1 signaling in response to amino acid deprivation (Fig 1A and 1B). Unlike the results of these downstream substrates, the phosphorylation levels of AKT, an upstream substrate of mTORC1, was similar between the patients and controls, suggesting that it was not affected by *SZT2* function (Fig 1A). To evaluate the response to amino acids, we next stimulated both cell lines with amino acid-containing medium for 10 min. In the patients’ cell lines, p-S6K and p-S6 levels were even more increased, while p-S6K and p-S6 levels remained low in the control cell lines (Fig 1A and 1B). The densitometric analysis also revealed that the phosphorylation levels of S6K and S6 in patient 2 were significantly higher than in patient 1, both under the starved and stimulated conditions, suggestive of more mTORC1 signaling activation in patient 2 (Fig 1B). To further evaluate the response to amino acids, we next re-stimulated the cell lines for 1 h and performed immunoblotting. After this long stimulation protocol, phosphorylation levels of S6K and S6 were increased in the control cell lines, whereas slight increase in the patients (Fig 1A). Densitometric analysis revealed that the difference in phosphorylation levels between the patients and the controls was not as marked as under starved and 10 min-stimulated conditions (Fig 1B). For easier interpretation of the results, a simplified molecular map is depicted in Fig 1C. Thus, our results suggest that the *SZT2* variants rendered mTORC1 signaling not only insensitive to amino acid deprivation, but also oversensitive to amino acid stimulation.

### Immunofluorescence

**Fig 2:**
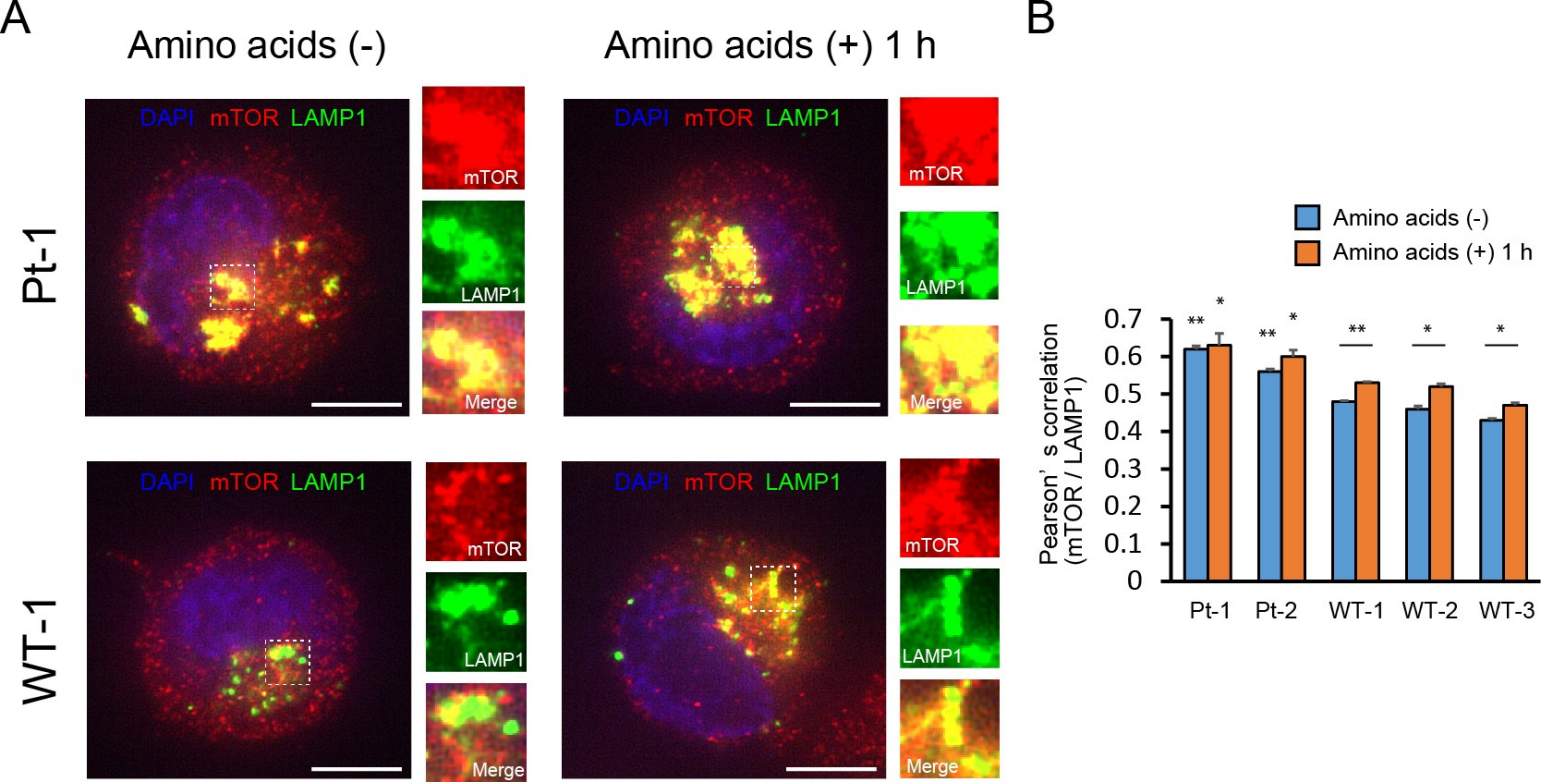
Amino acid-insensitive constitutive lysosomal localization of mTOR in the patients. (A) Cells were starved, or starved and re-stimulated with amino acids for 1 h. The localization of mTOR and LAMP1 was determined by immunofluorescence. Representative images of co-localization of mTOR (red) and LAMP1 (green) in patient 1 and wild type 1 are shown on behalf of each patient and control. Right panels show the enlarged view of co-localizing area. DAPI, nuclear staining (blue). Scale bars, 5 μm. See also S1 Fig for patient 2, wild type 2 and 3. (B) Quantification of co-localization between mTOR and LAMP1. Co-localization levels of patient 1 and 2 were significantly higher than those of controls under each condition. * *p* < 0.05. ** *p* < 0.01.

The activation of mTORC1 signaling correlates with the localization of mTORC1 [23]. When activated, mTORC1 moves from the cytoplasm to the lysosomal surface. To further examine whether mTORC1 signaling is activated in the patients, we analyzed the localization of mTOR and LAMP1, a well-characterized lysosomal marker, via immunofluorescence. This analysis revealed strong co-localization of mTOR and LAMP1 under amino acid-deprived conditions in the patients’ cell lines, while weak co-localization was observed in control cell lines. These results suggest constitutive mTORC1 localization at the lysosomal surface (Fig 2A and 2B and S1 Fig). After stimulation for 1 h, the co-localization levels remained significantly higher in the patients’ cell lines than in control cell lines, although co-localization increased in the control cell lines (Fig 2A and 2B and S1 Fig). These results showed that patients’ cell lines tended to be more sensitive to amino acids, consistent with the results of immunoblotting (Fig 2B).

## Discussion

In this study, we found that mTORC1 signaling is constitutively hyperactivated irrespective of nutrient status in patients with *SZT2* variants. Furthermore, this signaling pathway is easily activated with slight amino acid stimulation. To our knowledge, this is the first study to demonstrate functional consequence of *SZT2* mutations in humans.

KICSTOR is a key regulator of amino acid-dependent mTORC1 signaling [17]. When KICSTOR loses its function, GATOR1 is not able to interact with KICSTOR, and it is unable to inhibit mTORC1 activity, irrespective of the upstream signal (Fig 1C) [16, 17]. Consistent with these mechanisms, the patients’ cell lines showed increased p-S6K and p-S6 under amino acid-deprived conditions, suggesting amino acid insensitivity and constitutive activation of mTORC1 signaling. These results were validated by the amino acid-insensitive lysosomal localization of mTOR detected by immunofluorescence. Thus, the four *SZT2* variants the patients carried were demonstrated as loss-of-function mutations. The activation of mTORC1 leads to an increase in mRNA biogenesis, as well as translational initiation and elongation, resulting in cell growth and proliferation [20, 21]. mTOR functions as an important regulator of numerous neurological processes, including neural development and circuit formation [20]. Due to these mechanisms, hyperactivation of mTORC1 signaling can cause neurological diseases, such as developmental delay, macrocephaly, and epilepsy [2–6]. Although the pathomechanism in the patients’ neuronal cells remains to be directly examined, our results might reflect the functional alteration in the brain based on the fact that *SZT2* is expressed ubiquitously and the functional consequence of germline mutations in PI3K-AKT-mTOR pathway leading to hyperactivation of mTORC1 signaling has been determined using LCLs [4, 6].

As described above, functional alteration resulting from SZT2 deficiency has been discussed in the context of an amino acid-deprived condition [16, 17]. In contrast, it remains to be established how SZT2 deficiency affects the activation status of mTORC1 under the amino acid-replete condition. In this study, we focused on the amino acid-replete conditions and found that patients’ cell lines showed a higher tendency to activate mTORC1 in response to amino acids. Previous studies using HEK293T cell lines or mouse tissues were performed with 10 min stimulation with amino acids, which might have saturated the phosphorylation status of S6K and S6, and therefore little difference was observed between knockout and wild-type cells [16, 17]. Unlike these studies, our protocol with 10 min stimulation using LCLs was not sufficient for saturated phosphorylation, while 1 h stimulation rendered both patient and control cell lines toward saturated phosphorylation. Thus, shorter periods of stimulation with amino acids can highlight the functional difference between patients and controls. We considered that one of the reasons for this might be residual GATOR1 activity. In the patients, GATOR1 is not able to interact with KICSTOR because of a lack of SZT2, and subsequently mTORC1 is constitutively activated [16, 17]. With decreased baseline GATOR1 activity, amino acid-stimulation might easily inhibit GATOR1 and allow mTORC1 to be further activated. In contrast, full GATOR1 activity in the controls might not be inhibited by slight exposure to amino acids. Thus, patients’ cells were demonstrated to have a strong tendency toward hyperactivation of mTORC1 signaling, both under nutrient-replete and starved conditions. With these mechanisms, it seems to be reasonable that most of the patients with *SZT2* variants exhibit relatively severe neurological manifestations among the PI3K-AKT-mTOR pathway-related diseases [2–6].

Although most patients with *SZT2* variants exhibit severe phenotypes, some patients have been reported to exhibit mild or moderate phenotypic severity. In this respect, it has been considered that *SZT2* variants cause a broad phenotypic spectrum from epileptic encephalopathy and severe developmental delay to mild intellectual disability without epilepsy [8–11]. This variability has been assumed to depend on the residual activity of the SZT2 protein based on the fact that most of the mild or moderate cases carried non-null *SZT2* variants in at least one allele [10, 11]. Regarding the two patients in this study, their phenotypic severity differed. Moreover, patient 1 with a milder phenotype carried an intronic variant that was not comprehensively demonstrated to have a null effect, while patient 2 with a severe phenotype was considered to carry biallelic null variants leading to complete loss of SZT2 function [10, 13]. As expected, patient 2 showed higher phosphorylation levels of S6K and S6 under both deprived and 10 min-stimulated conditions, suggesting higher activation of mTORC1 (Fig 1A and 1B). Further research is needed to determine whether the residual SZT2 activity is associated with the phenotypic severity.

**Fig 3:**
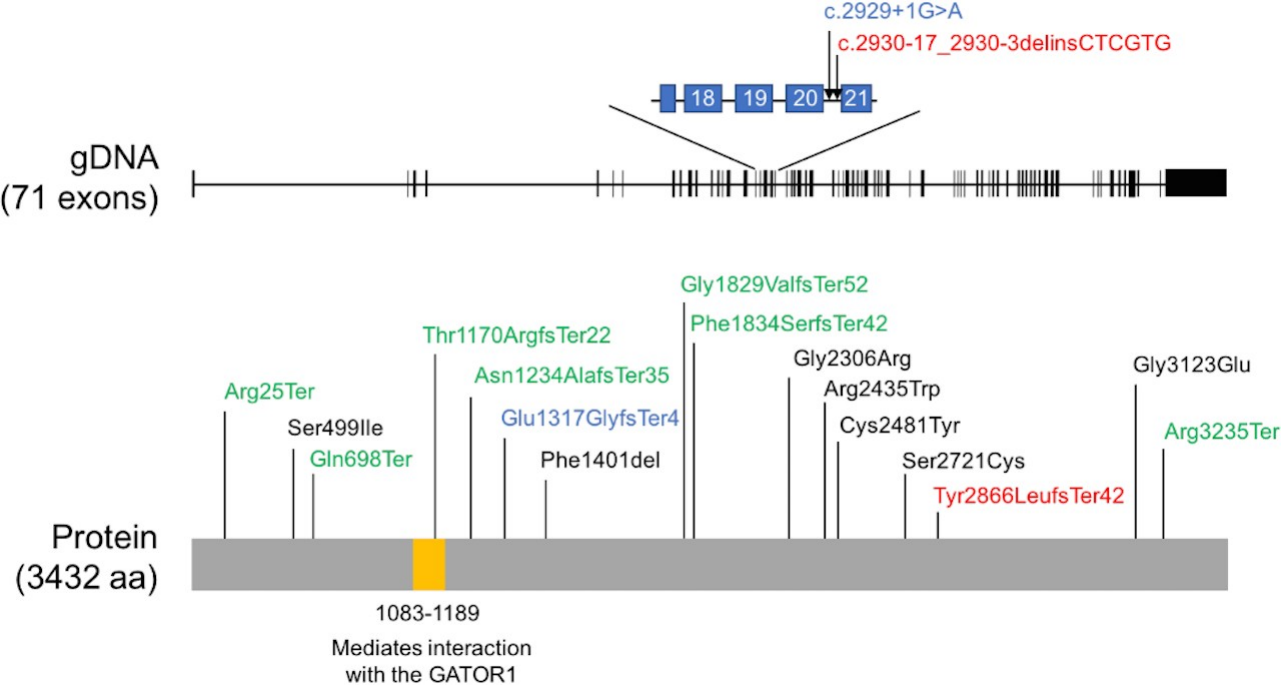
Localization of previously reported *SZT2* variants. Red and blue font indicates the variants in patient 1 and 2, respectively. Green font indicates null variants. Black font indicates missense or in-frame deletion variants.

To date, 18 *SZT2* variants have been reported (Fig 3) [7–15]. Among these variants, 9/18 are nonsense or frameshift variants, 2/18 are splice site variants, 6/18 are missense variants, and 1/18 is an in-frame deletion variant. The null variants, namely nonsense, frameshift, and splice site variants, were confirmed to result in hyperactivation of mTORC1 signaling in the current and previous studies [16, 17]. The missense and in-frame deletion variants, however, remain to be characterized. Because *SZT2* is a large gene containing 71 exons, increasing number of *SZT2* variants with unknown significance would be identified via next-generation sequencing. By the functional analysis we have established, pathogenicity of such newly identified variants can be determined, allowing for more detailed characterization of genotype-phenotype correlations.

In conclusion, we demonstrated that biallelic mutations in *SZT2* in two patients with a discernable neurodevelopmental disease induced hyperactivation of mTORC1 signaling in response to both amino acid starvation and stimulation, which provides new insights into the etiology of *SZT2*-related diseases. Further research should focus on how newly or previously identified variants with unknown significance affect SZT2 function.

## Supporting information

S1 Fig Constitutive lysosomal localization of mTOR in the patient 2. Representative images of co-localization of mTOR (red) and LAMP1 (green) in patient 2, wild type 2 and 3. Right panels show the enlarged view of co-localizing area. DAPI, nuclear staining (blue). Scale bars, 5 μm.
(TIF)
