# Supplementary figures and images for: Constitutive activation of mTORC1 signaling induced by biallelic loss-of-function mutations in SZT2 underlies a discernible neurodevelopmental disease
Source: PLoS One. 2019 Aug 20;14(8):e0221482. doi: 10.1371/journal.pone.0221482 (PMC6701784; doi:10.1371/journal.pone.0221482)

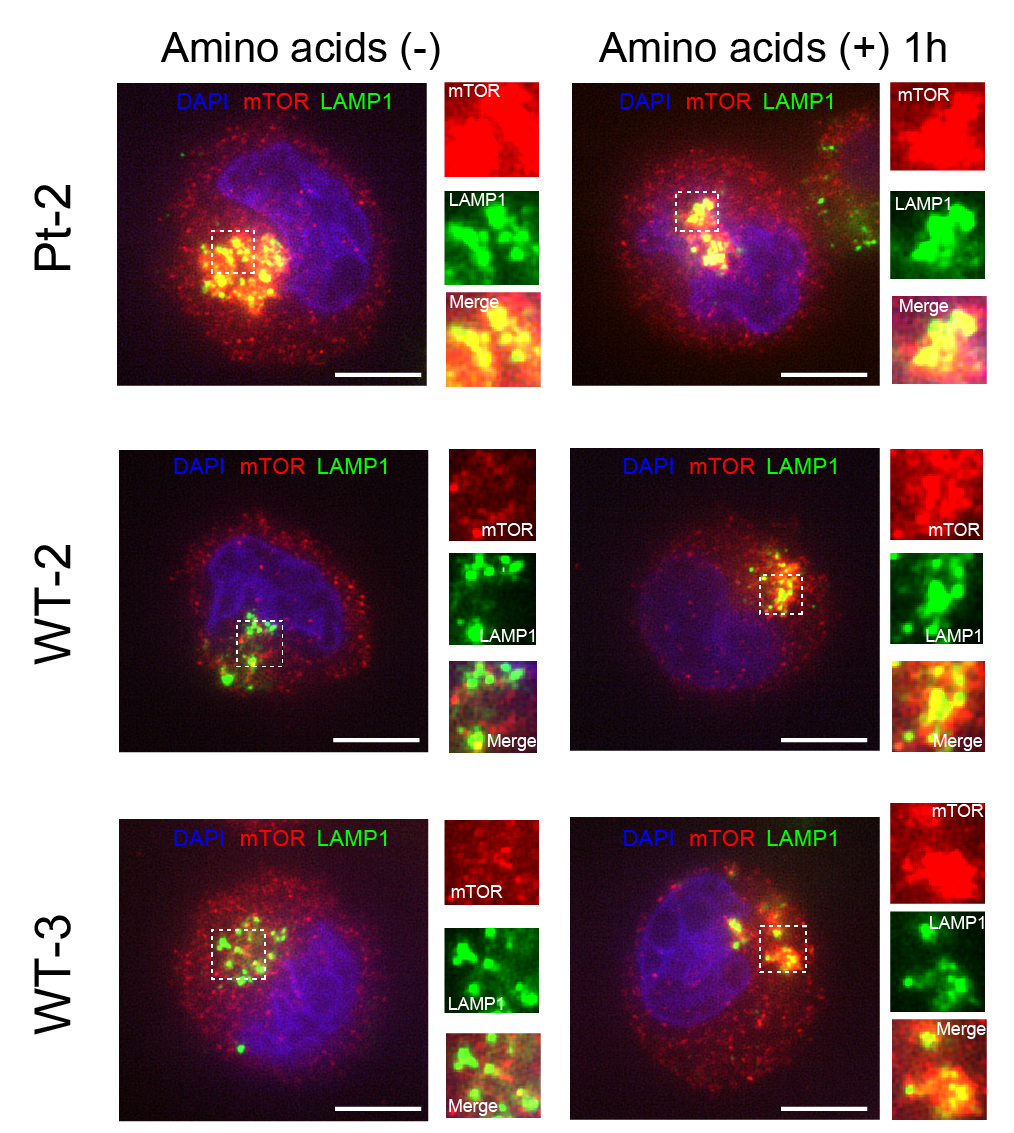

Supplement: S1 Fig — Representative images of co-localization of mTOR (red) and LAMP1 (green) in patient 2, wild type 2 and 3. Right panels show the enlarged view of co-localizing area. DAPI, nuclear staining (blue). Scale bars, 5 μm. (TIF) [file pone.0221482.s001.tif]
